# Staged pain or systemic crisis? Research on the employment effect of enterprise green transformation

**DOI:** 10.1007/s11356-023-29191-4

**Published:** 2023-08-25

**Authors:** Haixing Wang, Yaodong Zhou

**Affiliations:** School of Economics and Management, Beijing Jiaotong University, 100044 Beijing, China

**Keywords:** Green transformation, Employment effect, Dynamic non-linear relationship, Output effect, Factor complementary effect, Employment deprivation

## Abstract

The green transformation is a necessary path for industrialization to reach the advanced stage. Currently, achieving a win-win situation between green transformation and stable employment for residents has become a common concern for theoretical researchers and policymakers. This paper analyzes the influence mechanism of enterprise green transformation on employment using the partial equilibrium model. Furthermore, the employment effect and mechanism of enterprise green transformation are empirically verified with panel data from companies listed on the Shanghai and Shenzhen stock exchanges. The results indicate a dynamic non-linear relationship between enterprise green transformation and employment. As the transformation deepens, its impact on employment changes from negative to positive. Mechanism analysis reveals that enterprise green transformation has the potential to induce output growth and employment virtuous cycle. Additionally, environmental protection investment and labor employment in the transformation have a factor complementary effect. There is heterogeneity in the employment impact of enterprise green transformation, especially for production personnel, low-skilled labor, non-state-owned, heavy-polluting, and medium-low-tech enterprises, which experience stronger employment deprivation.

## Introduction

Employment is a crucial indicator of economic development and living standards, and it has always been a top priority for the government. As the world’s most populous developing country, China has placed great importance on employment and has maintained a low unemployment rate for a long time. However, due to the impact of the COVID-19 pandemic, the Russian-Ukraine conflict, the disruption of global supply chains, and the downward pressure on the economy, China’s employment situation is facing challenges and pressure while maintaining stability. In 2020, China emphasized increasing stability on six fronts and ensured job security as the first area of concern. Furthermore, the 2022 government work report places a prominent position on ensuring employment stability in the work plan. Therefore, stabilizing employment to ensure people’s livelihood and social stability is the current and future focus of the Chinese government’s work.

In the post-epidemic era, people have come to realize the crucial role of a healthy ecological environment in human survival. Governments worldwide are committed to promoting the green recovery of the global economy after the pandemic (García Vaquero et al. , 2021; Geels et al. , 2022), and industrial green transformation is one of the main directions of the green recovery. The experience of developed countries has shown that industrial green transformation has a positive impact on economic growth and environmental improvement, making it a necessary path to the advanced stage of industrialization. In recent years, the Chinese government has introduced related policies such as the “Industrial Green Development Plan (2016–2020)” and “The Fourteenth Five-Year Industrial Green Development Plan,” leading to remarkable results in industrial green development. As a critical subject and key actor in industrial green transformation, enterprise green transformation triggers systemic changes in the economy and society (Dechezleprêtre et al. , 2019). These changes include the adjustment of industrial structure, production methods, lifestyles, consumer behavior, as well as employment position and working style. However, the present research does not adequately address whether the green transformation has affected employment. On the one hand, much literature analyzes the impact of macro-environmental policies on employment from an institutional perspective, but the employment effect of enterprise micro-behavior is insufficiently studied. On the other hand, studies on green transformation mainly focus on assessing its economic and environmental effects (Yang , 2012; Miroshnychenko et al. , 2017; D’Angelo et al. , 2022; Aslam et al. , 2022), with little research assessing its social effects, especially the employment effect.

Some essential and urgent questions to be answered are whether the large-scale enterprise green transformation is shocking the employment market. What is the indirect impact of changes in output scale and environmental protection investment during the green transformation process on labor employment? Are there differences in the impact of green transformation on employment in heterogeneous employees and enterprises?

This paper offers three marginal contributions compared to the existing literature. Firstly, we demonstrate the dynamic changes in employment during the green transformation process from the perspective of a mathematical model based on the characteristics of enterprise green transformation stages and the green behavior choices of representative enterprises. Secondly, we determine whether an enterprise carries out green transformation and its degree based on five types of green behaviors, namely, green culture, green management, green innovation, green production, and green governance. This method indicates the comprehensive impact of various environmental policies on enterprises and accurately explores the labor market effect in the industrial green reform from the perspective of enterprise behavior. Thirdly, we conduct a deep analysis of the mechanism of green transformation, empirically testing the existence of two mechanisms: output effect and factor effect, and perform group tests on employee and enterprise heterogeneity. In particular, we innovatively use “the number of executives with environmental experience” as the instrumental variable to address potential endogeneity. These explorations offer empirical data and targeted advice for the formulation of more accurate industrial green policies, the prevention of risks associated with green reform, and the achievement of a “win-win” situation of green development and stable employment.

The structure of this paper is as follows: the “Literaturereview” section provides a review of the relevant literature. In the “Theoretical framework and hypotheses” section, we construct a mathematical model and propose research hypotheses. The “Research design” section introduces the selection of variables, data sources, and empirical model. The “Empirical results” section reports the empirical results and analysis, including stylized facts, baseline regression results, robustness tests, mechanism analysis, and heterogeneity analysis. Finally, we discuss the findings and implications in the “Conclusions and discussions” section.

## Literature review

Environmental policies are a crucial means for the government to promote the green transformation of economic development and adjust industrial structure towards sustainability. In recent years, the government has gradually increased its environmental regulations and intensively introduced a series of environmental policies, including environmental protection laws and regulations, clean energy policies, discharge permit systems, and carbon emissions trading schemes. The relationship between environmental policies and employment has been a longstanding focus of academic attention. Some literature holds that the transaction costs associated with environmental policies restrict enterprise production scales, which suppresses overall demand for labor, resulting in non-voluntary layoffs (Yip , 2018; Sheng et al. , 2019; Sheriff et al. , 2019; Raff and Earnhart , 2019; Lin and Li , 2021; Li et al. , 2022). Conversely, other literature has noticed the creation of new jobs resulting from environmental policies and the reallocation of labor cross-sector. This literature argues that environmental policies have a positive impact on the labor market. For example, Raff and Earnhart (2020) identified that environmental regulation policies create typical environmental jobs such as environmental enforcement officers and environmental supervisors. Bezdek et al. (2008) concluded that environmental protection not only promotes “environmental employment” but also increases standard jobs for accountants, engineers, computer analysts, clerks, factory workers, and truck drivers, with the latter accounting for a higher percentage. Yamazaki (2017) examined the impact of carbon tax reform in British Columbia from the perspective of labor cross-sector flow and indicated that the carbon tax reform reduced labor needs in carbon-intensive and trade-sensitive industries while increasing employment in clean service industries. On average, the total labor demand achieved a net increase of 0.74%. Hafstead and Williams III (2018) used a new general-equilibrium two-sector model to similarly confirm that environmental policies lead to the transfer of the labor force from polluting industries to non-polluting industries, and the net impact on overall employment is positive. Yoo and Heshmati (2019) demonstrated that labor will be reallocated from non-green to green sectors based on panel data of Korean manufacturing companies from 2004 to 2015. In addition, some literature from the perspective of environmental supervision level (Zhong et al. , 2021) and the dynamic impact of policy (Wang et al. , 2020) have proven that the relationship between environmental policies and employment presents non-linear characteristics.

In summary, there is no consistent conclusion on the impact of environmental policies on employment. The strong correlation between environmental policies may result in greater policy concentration, making it difficult to identify the net influence of a single policy on employment. Furthermore, environmental policies may deprive employment opportunities in different proportions (Yip , 2018; Liu et al. , 2021), and different study objects may cause differences in labor demand for policy (Lin and Li , 2021). For instance, if the study object is dominated by regulatory industries such as polluting industries, energy industries, and manufacturing enterprises, the results usually overestimate the damage caused by environmental policies (Hafstead and Williams III , 2018; Yoo and Heshmati , 2019).

Enterprises incorporate environmental protection into their development strategies and carry out a series of green activities, which can reduce environmental pollution penalties, improve their reputation (Griskevicius et al. , 2010), and attract environmentally friendly consumers to make purchase decisions (Peattie , 2001). Therefore, in pursuit of economic profits, macro-environmental policies act as institutional arrangements that prompt enterprises to take green measures and carry out green transformation (Miroshnychenko et al. , 2017; Shen et al. , 2020; D’Angelo et al. , 2022; Aslam et al. , 2022). The existing literature has explored the impact of enterprise green behavior on the labor market. Based on questionnaires from five European countries, Getzner (2002) confirmed that the adoption of clean technologies by enterprises can significantly increase the scale and quality of employment. Gagliardi et al. (2016) investigated the link between environmentally friendly technological change and job creation at the firm level and found a positive impact of green innovation on long-run job creation. Liu et al. (2018) used data from Chinese manufacturing enterprises from 2001 to 2007 to estimate the impact of corporate pollution prevention strategies on labor demand. The study showed that pollution control negatively affects employment scale, and further disaggregating abatement strategies revealed that the employment promotion effect of “end-pipe” is not enough to offset the crowding-out effect of “changes in process.”

To sum up, the existing literature has only considered the impact of specific green behaviors on employment, with inconsistent conclusions. It is worth noting that a single green behavior cannot accurately reflect the actual effects of enterprise green transformation under multiple environmental policies, leading to estimation bias. Moreover, there is a significant gap between the realization of green transformation in different countries (Cheba et al. , 2022). The institutional environment and labor market in developing countries is relatively imperfect, making it difficult to fully apply the advanced experience of developed countries to developing countries. Therefore, in this study, we take China as an example of a developing country in the accelerated industrialization stage. Based on the perspective of enterprise behavior, we adopt comprehensive green measures to express green transformation and analyze its impact and mechanism on employment.

## Theoretical framework and hypotheses

1. We assume that the national economy has *I* industries, with each industry denoted by *i*. ${Y_{it}}$ represents the output of industry *i* in year *t*. We follow the research methods of Sanz and Schwartz (2013) and introduce the pollution intensity index into production functions. The production function ${Y_{it}}$ is influenced by the labor factor input ${L_{it}}$, the capital factor input ${K_{it}}$, and the pollution intensity index ${Z_{it}}$ ($0 \le {Z_{it}} \le 1$). The coefficients of output elasticity for labor and capital are denoted by $\alpha$ and $\beta$, respectively, and satisfy $0< \alpha < 1$ and $0< \beta < 1$. The parameter $\sigma$ indicates the cleanliness of the technology used in production, where $\sigma > 0$ indicates the use of polluting technology and $\sigma < 0$ indicates the use of green technology. A smaller value of $\sigma$ indicates a higher level of green technology.
2. We assume that the capital input factors are divided into three categories. The first category is the traditional fixed asset input necessary for enterprise production ($K_{it}^F$). The second category is the investment in special equipment for pollution control ($K_{it}^E$). The third category is the investment in special equipment needed for technological progress ($K_{it}^I$).
3. We assume that the product price and the price of production factors remain unchanged. ${P_i}$ represents the product price of the representative enterprise in industry *i*, and ${W_i}$ and $R_i^j$ represent the labor wage and capital interest, respectively. The unit pollution control cost is a function of the technology level $\sigma$, denoted as ${C_i} = {e^\sigma }$ ($\sigma > 0$).
4. We assume that the production function of the representative enterprise is the Cobb-Douglas function.

Based on the above assumptions, we compare the labor employment changes of enterprises under the following three scenarios:

1. The base period of green transformation is not considered ($t = 0$).

Assuming that the enterprise has not carried out the green transformation, ${Z_{it}} = 1$ at this time, indicating that the enterprise does not consider environmental pollution to bring output loss. Additionally, the enterprise does not provide financial support for pollution control and the advancement of green technology. Therefore, the production function of the representative enterprise is

1 $$\begin{aligned} {Y_{i0}} = {({L_{i0}})^\alpha }{(K_{i0}^F)^{{\beta _F}}} \end{aligned}$$

Its profit function is as follows:

2 $$\begin{aligned} {\pi _{i0}} = {P_i}{({L_{i0}})^\alpha }{(K_{i0}^F)^{{\beta _F}}} - {W_i}{L_{i0}} - R_{i0}^FK_{i0}^F \end{aligned}$$

When the representative enterprise maximizes its profit, the labor demand can be expressed as

3 $$\begin{aligned} \ln {L_{i0}}= & {} \frac{1}{{1 - \alpha }}\ln \alpha + \frac{1}{{1 - \alpha }}\ln {P_i} + \frac{{{\beta _F}}}{{1 - \alpha }}\ln K_{i0}^F\nonumber \\{} & {} - \frac{1}{{1 - \alpha }}\ln {W_i} \end{aligned}$$

Based on (3), the factors that affect labor employment in the base period include price, fixed assets, and wages.

2. In the initial stage of green transformation with terminal pollution control ($t=1$).

Enterprises prioritize environmental concerns ($Z_{it}<1$), and the use of polluting technology ($\sigma >0$) in the production process leads to output losses due to pollution emissions. During this stage, enterprises have two investment options for green transformation: increasing investment in fixed assets of pollution control equipment such as scrubbers and precipitators to improve the level of terminal treatment of pollutants, or increasing investment in hardware equipment for technology research and development (R &D) such as research and development centers and professional laboratories. In reality, government regulatory departments primarily evaluate the effectiveness of enterprise green transformation based on pollution emission reduction. Moreover, the amount of capital required for technological R &D investment is relatively high. Therefore, it is assumed that enterprises in the early stage of green transformation will prioritize increasing investment in terminal pollution control equipment. The production function of the representative enterprise is as follows:

4 $$\begin{aligned} {Y_{i1}} = {({L_{i1}})^\alpha }{(K_{i1}^F)^{{\beta _F}}}{(K_{i1}^E)^{{\beta _E}}}Z_{i1}^\sigma \end{aligned}$$

Its profit function is as follows:

5 $$\begin{aligned} {\pi _{i1}} = {P_i}{Y_{i1}} - {W_i}{L_{i1}} - R_{i1}^FK_{i1}^F - R_{i1}^EK_{i1}^E - {C_i}Z_{i1}^\sigma {Y_{i1}} \end{aligned}$$

When the representative enterprise maximizes its profit, the labor demand can be expressed as

6 $$\begin{aligned} \ln {L_{i1}}= & {} \frac{1}{{1 - \alpha }}\ln \alpha \!+ \underbrace{\frac{1}{{1 - \alpha }}\ln ({P_i} \!- {e^\sigma }Z_{i1}^\sigma )}_{(-) } \!+ \frac{{{\beta _F}}}{{1 - \alpha }}\ln K_{i1}^F\nonumber \\{} & {} + \underbrace{\frac{{{\beta _E}}}{{1 - \alpha }}\ln K_{i1}^E}_{ (+) } + \underbrace{\frac{\sigma }{{1 - \alpha }}\ln Z_{i1}}_{(-) } - \frac{1}{{1 - \alpha }}\ln {W_i} \end{aligned}$$

Compared to (3), the labor demand of enterprises is influenced by new variables in the green transformation stage. Firstly, the cost of pollution control reduces the relative price of products, which indirectly lowers labor demand. Secondly, investment in fixed assets for pollution control equipment increases labor demand. Finally, the increased treatment costs of polluting production technologies inhibit labor demand.

We further analyze the impact of technological progress on labor demand in the early stage. The result is as follows:

7 $$\begin{aligned} \begin{aligned} \partial \ln {L_{i1}}/\partial \sigma = \frac{{{P_i} - {e^\sigma }Z_{i1}^\sigma (1 + 2\ln {Z_{i1}})}}{{1 - \alpha ({P_i} - {e^\sigma }Z_{i1}^\sigma )}} \end{aligned} \end{aligned}$$

As shown in (7), the impact of technological progress on labor demand is uncertain and depends on the pollution emission intensity (${Z_{it}}$) of enterprises. When the pollution emission intensity of enterprises is high (${Z_{it}} < {e^{ - 0.5}}$), $\partial \ln {L_{i1}}/\partial \sigma > 0$, indicating that technological progress inhibits labor demand. Conversely, when the pollution emission intensity of enterprises is low (${Z_{it}} > {e^{ - 0.5}}$), $\partial \ln {L_{i1}}/\partial \sigma < 0$, indicating that technological progress promotes labor demand. This suggests that the impact of technological progress on the labor demand of enterprises with different pollution levels differs significantly, with enterprises having higher pollution levels experiencing greater employment losses in the process of green transformation. Therefore, the labor employment change of enterprises in the early stage of green transformation is uncertain.

3. In the deepening stage of green transformation with technological progress ($t=2$).

It is assumed that enterprises continue to prioritize environmental concerns (${Z_{it}}<1$). The production technology is changed from polluting technology ($\sigma >0$) to green technology ($\sigma <0$). Green technology improves production efficiency and promotes output growth, while also solving the problem of pollutants associated with the production process. As a result, the cost of pollution control is assumed to be 0. Additionally, it is assumed that enterprises increase investment in technology R &D hardware equipment on top of the investment made in period 1. The production function of the representative enterprise is as follows:

8 $$\begin{aligned} \begin{aligned} {Y_{i2}} = {({L_{i2}})^\alpha }{(K_{i2}^F)^{{\beta _F}}}{(K_{i2}^E)^{{\beta _E}}}{(K_{i2}^I)^{{\beta _I}}}Z_{i2}^\sigma \end{aligned} \end{aligned}$$

Its profit function is as follows:

9 $$\begin{aligned} \begin{aligned} {\pi _{i2}} = {P_i}{Y_{i2}} - {W_i}{L_{i1}} - R_{i1}^FK_{i1}^F - R_{i1}^EK_{i1}^E - R_{i1}^IK_{i1}^I \end{aligned} \end{aligned}$$

When the representative enterprise maximizes its profit, the labor demand can be expressed as

10 $$\begin{aligned} \ln {L_{i2}}= & {} \frac{1}{{1 - \alpha }}\ln \alpha + \underbrace{\frac{1}{{1 - \alpha }}\ln {P_i}}_{( + )} + \frac{{{\beta _F}}}{{1 - \alpha }}\ln K_{i2}^F\nonumber \\{} & {} + \frac{{{\beta _E}}}{{1 - \alpha }}\ln K_{i2}^E + \underbrace{\frac{{{\beta _I}}}{{1 - \alpha }}\ln K_{i2}^I}_{( + )} + \underbrace{\frac{\sigma }{{1 - \alpha }}\ln Z_{i2}}_{( + )}\nonumber \\{} & {} - \frac{1}{{1 - \alpha }}\ln {W_i} \end{aligned}$$

Compared to (6), the labor demand of enterprises has undergone new changes in the deepening stage of green transformation with technological progress. Firstly, investment in technology R &D hardware equipment increases labor demand. Secondly, technological progress avoids employment losses in the process of pollutant emission and treatment by solving the pollutant problems associated with production. We further analyze the impact of technological progress on labor demand during the deepening transformation period. The result is as follows:

11 $$\begin{aligned} \begin{aligned} \partial \ln {L_{i2}}/\partial \sigma = \frac{{\ln {Z_{i2}}}}{{1 - \alpha }} < 0 \end{aligned} \end{aligned}$$

As shown in (11), during the deepening period of green transformation, technological progress is conducive to increasing labor demand. This suggests that technological progress can create employment. Combining (10) and (11), it can be seen that during the deepening period of green transformation, investment in technology R &D hardware and technological progress jointly promote the growth of labor demand. Therefore, we can put forward Hypothesis 1.

**Hypothesis 1.** There is a dynamic non-linear relationship between enterprise green transformation and employment, and the impact on employment changes from negative to positive as the transformation deepens.

The output effect of enterprise green transformation refers to the employment changes caused by changes in production. However, the impact of enterprise green transformation on the production scale is uncertain. Firstly, according to the compliance cost hypothesis, the green transformation process may increase pollution control and transaction costs, squeezes out productive investment and innovation activities, and force out backward capacity that cannot meet environmental standards. This can lead to a reduction in output. Secondly, according to the innovation offset effect, rational producers may enhance green technology innovation and choose cleaner production and pollution control technologies to maximize profits. This improvement in technology can reduce the unit cost of pollution control and avoid the output crowding-out effect caused by rising production costs. Meanwhile, the product price reduction caused by technological innovation makes products more competitive, which encourages enterprises to recruit more labor to expand production (Zhu et al. , 2021). Additionally, the expansion of new product markets generated by technological innovation can create new employment opportunities (Harrison et al. , 2014). Thus, the enterprise green transformation affects employment by influencing output scale, but there is uncertainty about the specific effect.

The factor effect of enterprise green transformation refers to the employment changes caused by environmental protection investment. The green transformation through environmental investment is the reflection of enterprise social responsibility (Wang et al. , (2018), which is beneficial to enhance the reputation and value of the enterprise (De Blas , 2021). Hence, enterprises often choose to increase environmental protection investment to improve the level of green transformation (Liu et al. , 2022). However, the employment effect of environmental protection investment is uncertain and depends on whether it has a substitution or complementary effect. On the one hand, environmental protection investment is used for environmental protection such as the purchase of advanced environmental protection equipment, the reform of energy-saving and environmental protection, and pollution control. The installation, operation, and maintenance of equipment extend the production chain and related processes, which contributes to employment and forms the factor complementary effect. On the other hand, environmental protection equipment can improve labor productivity by optimizing the production process, which reduces labor demand and forms the factor substitution effect. Based on these, we propose the following hypothesis.

**Table 1:** Enterprise green transformation index construction

Dimension	Green measures
Green culture	1. Environmental protection concepts
	2. Environmental protection goals
	3. Environmental education and training
	4. Environmental honors or rewards
Green management	1. Environmental management institutional system
	2. Emergency mechanism for environmental events
	3. Special environmental protection actions
	4. ISO14001 certification
Green innovation	1. Green patents
Green production	1. Clean production processes
	2. Reduction of waste gas, waste water, waste residue and greenhouse gas emissions
	3. “Three simultaneous” system
Green governance	1. Pollutant emissions meet the standards
	2. Sudden environmental accidents
	3. Environmental violations events
	4. Environmental petition events

**Hypothesis 2.** The enterprise green transformation affects employment through both the output effect and the factor effect.

## Research design

### Variable selection

#### Dependent variable

The dependent variable of this paper is labor employment, represented by the natural logarithm of employees of listed companies ($\ln L$).

#### Independent variables

The independent variable in this study is the green transformation of enterprises, denoted as *GT*. Enterprises in China do not have specific requirements to publicly disclose their environmental information, resulting in greater discretion in the content, form, and quality of disclosure. Current literature typically utilizes green total factor productivity or green patents as measures of green transformation. Considering the lack of the above methods, we refer to Peng et al. (2022) to construct the comprehensive index of green transformation from the perspective of enterprise behavior.

Based on the annual reports, social responsibility reports, and sustainability reports of listed companies, we use text analysis to express green transformation from green culture, green management, green innovation, green production, and green governance. It is shown in Table 1. (1) Green culture refers to an enterprise’s commitment to environmentally friendly development, as evidenced by establishing environmental concepts and goals, conducting environmental education and training, and receiving environmental honors awards. (2) Green management concerns the implementation of environmentally friendly measures at the institutional level (Xing et al. , 2020), including establishing an environmental management institutional system, setting up an emergency mechanism for environmental events, carrying out special environmental protection measures, and obtaining ISO 14001 certification. (3)Green innovation involves the creation of new designs and products that reduce or eliminate the use and production of harmful substances, as generally measured by green patents (Berrone et al. , 2013; Chen et al. , 2021). (4) Green production involves both front-end and end-pipe management of the production process (Chiou et al. , 2011), including clean production processes, reduction of waste gas, waste water, waste residue, and greenhouse gas emissions, and the “three simultaneous” system. (5) Green governance reflects an enterprise’s pollution control effectiveness, including whether pollutant emissions meet standards, the occurrence of sudden environmental accidents, and environmental violations and petitions. We assign a value of 1 to each dimension if the enterprise implements at least one green measure in that dimension; otherwise, the value is 0. The sum of each dimension is the green transformation score ($0 \le GT \le 5$), where a higher value indicates a greater degree of green transformation.

#### Control variables

We refer to Xing et al. (2020) and Liu et al. (2022) to select nominal wage($\ln W$), asset-liability ratio(*Lev*), growth ability(*Growth*), profitability(*Roa*), capital intensity (*Intensity*), shareholding concentration(*Top*1), enterprise size(*Size*), age of listing(*Age*), agency costs(*Agencost*), and cash flow(*Cflow*) as the firm-level control variables. The definitions and descriptions of variables are presented in Table 2.

**Table 2:** Control variables definition

Variables	Symbol	Definition
Nominal wage	$\ln W$	The natural logarithm of cash paid to employees
		(including employee salary, social insurance, housing
		fund, etc.)
Asset-liability ratio	*Lev*	Total liabilities divided by total assets
Growth ability	*Growth*	The percentage increase in operating revenue
Profitability	*Roa*	Net profit divided by average total assets
Capital intensity	*Intensity*	The net value of fixed assets divided by total assets
Shareholding		
concentration	*Top*1	The percentage ownership of the largest shareholder
Enterprise size	*Size*	The natural logarithm of total assets
Age of listing	*Age*	The natural logarithm of the listing’s age plus one
Agency costs	*Agencost*	Administrative expense divided by operating revenue
Cash flow	*Cflow*	The ratio of cash flow from operations to total assets

**Fig. 1:**
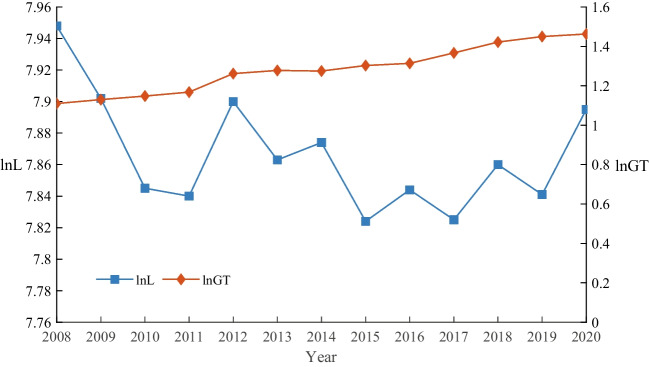
The trend of enterprise green transformation and employment

### Data sources

We collected data on Chinese A-share listed companies from 2008 to 2020 using the CSMAR database. To avoid the effect of abnormal samples, the following companies are excluded from the sample: (1) companies designated “ST,” “PT,” and “*ST” during the sample period; (2) financial companies; (3) companies with serious deficiencies in financial data. After the above processing, we finally obtain unbalanced panel data including 2186 listed companies and a total of 16,050 observations.The sample consists of a total of 39 industries, including mining, manufacturing, electricity, heat, gas, and water production and supply.

### Model

To account for employment rigidity, we have incorporated the lagged term of the dependent variable into our dynamic panel model, which is presented below.

12 $$\begin{aligned} \ln {L_{i,t}}= & {} {\beta _0} + {\beta _1}\ln {L_{i,t - 1}} + {\beta _2}\ln G{T_{i,t}} + \beta _{3}\ln GT_{i,t}^2 \nonumber \\{} & {} + \beta _{j}{x_{i,t}} + \delta _{i} + \varepsilon _{it} \end{aligned}$$

In the above model, ${\delta _i}$ represents the unobserved effect of enterprise *i*, while ${\varepsilon _{it}}$ denotes the random error. To address potential endogeneity issues stemming from the lagged term of the dependent variable, as well as the non-consistency of parameter results associated with the fixed effects approach, we employ difference-GMM and system-GMM for estimation.

## Empirical results

### Stylized facts

Figure 1 illustrates the trends in green transformation and employment.The average value of green transformation increased from 1.11 to 1.46 between 2008 and 2020, suggesting an upward trend in overall enterprise green transformation. To some extent, this confirms the effectiveness of the green governance concept after 2005. The average value of labor force employment in enterprises exhibits an initial decline followed by a subsequent rise. Besides the impact of the 2008 financial crisis and the inflection point of China’s demographic dividend in 2011, further empirical verification is needed to determine if the green transformation has exacerbated this trend. Simultaneously, the presence of consistent or opposing trends between green transformation and employment scale suggests a potential nonlinear relationship.Therefore, it is necessary to introduce quadratic terms and use dynamic panel models to scientifically analyze the impact of green transformation on employment.

**Table 3:** The impact of enterprise green transformation on employment

Variables	(1)	(2)	(3)	(4)
Method	DGMM	SGMM	DGMM	SGMM
$\ln GT$	$-$0.038**	$-$0.027**	$-$0.885***	$-$0.682***
	($-$2.45)	($-$1.99)	($-$3.44)	($-$3.45)
$\ln (GT)^{2}$			0.346***	0.275***
			(3.26)	(3.40)
$\ln L( - 1)$	0.406***	0.314 ***	0.437***	0.448***
	(31.99)	(34.33)	(56.76)	(79.28)
$\ln W$	$-$0.067***	$-$0.411***	$-$0.025***	$-$0.032***
	($-$2.74)	($-$26.55)	($-$6.54)	($-$11.15)
*Lev*	0.192***	0.354***	0.166	0.025
	(3.98)	(12.30)	(1.53)	(0.44)
*Growth*	0.001***	0.002***	0.001*	0.001**
	(6.31)	(16.72)	(1.72)	(2.12)
*Roa*	0.020**	0.056***	0.004	0.031***
	(2.31)	(17.11)	(0.17)	(9.87)
*Intensity*	$-$0.156**	$-$0.151***	$-$0.051	$-$0.176**
	($-$2.58)	($-$4.13)	($-$0.36)	($-$2.50)
*Top*1	0.312***	$-$0.057	0.160	$-$0.047
	(3.45)	($-$1.02)	(0.82)	($-$0.44)
*Size*	0.472***	0.267***	0.014	0.032**
	(27.77)	(28.64)	(0.56)	(2.47)
*Age*	0.078***	0.107***	0.091**	0.002
	(4.97)	(12.90)	(2.21)	(0.12)
*Agencost*	$-$0.035***	$-$0.032***	$-$0.232***	$-$0.122***
	($-$5.14)	($-$6.01)	($-$11.03)	($-$12.30)
*Cflow*	$-$0.142***	$-$0.067**	$-$0.335***	$-$0.123**
	($-$4.12)	($-$2.44)	($-$3.94)	($-$1.98)
$\_cons$		$-$8.978***		4.288***
		($-$53.64)		(15.76)
Year FE	Yes	Yes	Yes	Yes
AR(1)	$-$7.56	$-$8.17	$-$8.35	$-$9.69
	(0.000)	(0.000)	(0.000)	(0.000)
AR(2)	0.68	$-$0.36	1.14	1.10
	(0.498)	(0.718)	(0.255)	(0.272)
sargan	70.55	137.35	124.17	120.35
	(0.624)	(0.345)	(0.285)	(0.301)
*N*	11,713	13,815	11,713	13,815

***, **, * denote significance at 1%, 5%, and 10%, respectively. Z-statistics are reported in parentheses. The Sargan test is used to test the over-identification constraint for GMM estimation. AR(1) and AR(2) are used to examine first- and second-order autocorrelation of disturbance terms, respectively

**Table 4:** A series of robustness tests

Variables	(1)	(2)	(3)	(4)
$\ln GT$	$-$0.542*	$-$3.342*	$-$0.772**	$-$0.429*
	($-$1.83)	($-$1.87)	($-$2.19)	($-$1.68)
$\ln (GT)^2$	0.255**	1.341*	0.251*	0.144*
	(2.20)	(1.93)	(1.83)	(1.65)
$\ln L( - 1)$	0.746***	0.060***	0.728***	0.796***
	(20.19)	(2.67)	(11.85)	(15.97)
$\ln W$	$-$0.225***	$-$0.080*	$-$0.216***	$-$0.141***
	($-$4.89)	($-$1.71)	($-$3.79)	($-$3.28)
*Lev*	0.075**	0.410**	0.104***	0.069***
	(2.41)	(2.38)	(3.90)	(3.33)
*Growth*	0.000	0.000***	0.000***	0.000***
	(1.33)	(9.30)	(7.52)	(8.14)
*Roa*	0.002	1.654***	1.654***	0.056***
	(0.14)	(5.05)	(3.53)	(3.53)
*Intensity*	$-$0.161	0.132	0.034	$-$0.040
	($-$0.97)	(0.16)	(0.93)	($-$1.35)
*Top*1	$-$0.008	$-$0.580***	$-$0.089***	$-$0.060**
	($-$0.22)	($-$4.27)	($-$2.72)	($-$2.12)
*Size*	0.283***	$-$0.093	0.060***	0.055***
	(6.23)	($-$0.65)	(5.77)	(6.62)
*Age*	0.001	0.363***	0.068***	0.060***
	(0.16)	(9.21)	(11.18)	(12.78)
*Agencost*	0.090	$-$1.420***	0.132	$-$0.159**
	(1.31)	($-$2.87)	(1.15)	($-$1.97)
*Cflow*	$-$0.139**	$-$0.468	$-$0.005	$-$0.012
	($-$2.05)	($-$1.04)	($-$0.10)	($-$0.28)
$\_cons$	0.488**	5.64***	$-$3.027***	$-$2.117***
	(2.00)	(4.05)	($-$4.64)	($-$4.59)
Year FE	Yes	Yes	Yes	Yes
AR(1)	$-$8.40	$-$12.10	$-$13.26	$-$10.89
	(0.000)	(0.000)	(0.000)	(0.000)
AR(2)	0.98	$-$0.02	0.42	0.48
	(0.325)	(0.984)	(0.676)	(0.632)
sargan	80.83	128.99	89.78	84.73
	(0.578)	(0.338)	(0.117)	(0.207)
*N*	13,815	6000	13,491	12,272

***, **, * denote significance at 1%, 5%, and 10%, respectively. Z-statistics are reported in parentheses. The dependent variables for columns (1) and (2) are $\ln RL$ and $\ln CL$, respectively. The dependent variables for columns (3) and (4) are $\ln L$

### Baseline regression results

Table 3 presents the regression results of the impact of enterprise green transformation on employment. The coefficient of $\ln GT$ is $-$0.038 in column (1) and $-$0.027 in column (2), and both are statistically significant at the 5% level. After introducing the quadratic term, the coefficient of $\ln GT$ in column (3) and column (4) remains significantly negative, while the coefficient of $\ln (GT)^{2}$ is significantly positive. This confirms the existence of a dynamic non-linear relationship between green transformation and employment, displaying a U-shaped pattern overall. The impact of green transformation on employment changes from negative to positive as the degree of transformation deepens. The turning points of the U-shaped curve for green transformation are 1.28 and 1.24, respectively. This suggests that enterprises can effectively avoid employment losses and achieve employment growth in the transformation process only when they adopt more than three types of green measures. Indeed, the multi-dimensional, multi-measure, and multi-directional transformation exploration of enterprises in areas such as green culture, green management, green innovation, green production, and green governance plays a vital role in mitigating employment deprivation and fostering employment growth.

### Robustness tests

#### Replace the dependent variable

We consider relative employment ($\ln RL$) and employment change ($\ln CL$) as alternative variables to employment ($\ln L$). Relative employment is expressed as the natural logarithm of employees per 10,000 yuan of total assets, while the employment change is equal to the difference between the natural logarithm of employees in the current period and the previous period. The regression results for these alternative variables are presented in columns (1) and (2) of Table 4.^1^ The coefficients of $\ln GT$ are $-$0.542 and $-$3.342, respectively, and the coefficients of $\ln (GT)^{2}$ are 0.255 and 1.341, respectively. All coefficients pass the significance test, which is consistent with the baseline regression results.

**Table 5:** Instrumental variable estimation results

The first stage estimation		
Variables	$\ln GT$	$\ln (GT)^2$
*number*	0.128*** (5.80)	0.969*** (5.50)
$number^2$	$-$0.003* ($-$1.72)	$-$0.017* ($-$1.75)
The second stage estimation		
Variables	$\ln L$	
$$\ln \, GT$$	$$-2.670 **\, (-2.01)$$	
$\ln (GT)^2$	0.279* (1.65)	
Hausman test	135.15 (*p*=0.000)	
*number*, *F*-value	20.88	
$numbe^2$, *F*-value	20.53	
AR test	67.15 (*p*=0.000)	
Wald test	22.92 (*p*=0.000)	
Control variables	Yes	
Year FE	Yes	
*N*	13,815	

***, **, * denote significance at 1%, 5%, and 10%, respectively. Z-statistics are reported in parentheses

#### Truncated regression

To exclude the effect of abnormal values, we truncate the employment and enterprise green transformation data at the 1% and 5% levels. The results of the truncated regression are displayed in columns (3) and (4) of Table 4. The coefficients of $\ln GT$ are $-$0.772 and $-$0.429, respectively, and the coefficients of $\ln (GT)^{2}$ are 0.251 and 0.144, respectively. All coefficients pass the significance test, demonstrating the robustness of the baseline regression results.

#### Endogenous treatment

The baseline regressions may overlook some variables that influence green transformation and employment, resulting in endogeneity issues. Therefore, it is necessary to use the instrumental variable (IV) approach to address endogeneity. Executives are the key decision-makers in business, and their decision-making behavior is influenced by prior experience and background (Hoffman and Ocasio , 2001). Executives with environmental experience^2^ are more aware of environmental protection, strictly adhere to relevant national environmental policies, and actively respond to the green development strategy. Consequently, they are more motivated to promote enterprise green transformation, which satisfies the requirement of instrumental variable correlation. Additionally, the environmental experience of executives is not directly related to employment, meeting the condition of instrumental variable exogeneity. Finally, to avoid potential multicollinearity issues associated with the quadratic term of the core explanatory variable in the model, we have chosen the number of executives with environmental experience (*number*) and its quadratic term ($number^2$) as instrumental variables.

Next, we assess the effectiveness of the instrumental variables. Firstly, the endogeneity test shows that the Hausman test rejects the original hypothesis of “all explanatory variables are exogenous,” indicating that the model has endogenous explanatory variables. Secondly, the weak instrumental variable test indicates that the *p*-values of the AR test (67.15) and Wald test (22.92) are less than 0.01, while the *F*-value of instrumental variables is greater than 10. This suggests that there is no weak instrumental variable problem in the model. Therefore, it is appropriate to use the number of executives with environmental experience and its quadratic term as the instrumental variables. The regression results of the instrumental variable approach are presented in Table 5. The first stage of estimation shows a significant effect of instrumental variables on endogenous variables. The second stage results demonstrate a U-shaped relationship between green transformation and employment. Compared with the baseline regression, the coefficients estimated by 2SLS show only small changes, indicating that the endogeneity problems have little effect on the estimation results of this study.

**Table 6:** Mechanism analysis

Variables	Output effect		Factor effect			
	(1)	(2)	(3)	(4)	(5)	(6)
$\ln GT$	$-$0.150**	$-$0.530**	0.287**	$-$0.951***	0.144***	$-$0.910**
	($-$2.19)	($-$2.34)	(2.05)	($-$3.05)	(4.09)	($-$2.33)
$\ln (GT)^2$	0.146***	0.227**	$-$0.022	0.342***	$-$0.003	0.305**
	(5.19)	(2.49)	($-$1.31)	(2.92)	($-$0.74)	(2.03)
$\ln output$		0.066***				
		(6.74)				
$\ln EP{I_1}$				0.017*		
				(1.81)		
$\ln EP{I_2}$						0.028***
						(6.57)
$\_cons$	1.167***	$-$2.200***	$-$0.247	0.564	2.925***	$-$2.096***
	(9.41)	($-$4.65)	($-$0.71)	(1.48)	(10.25)	($-$2.82)
Control	Yes	Yes	Yes	Yes	Yes	Yes
variables						
Year FE	Yes	Yes	Yes	Yes	Yes	Yes
AR(1)		$-$9.48		$-$4.32		$-$8.70
		(0.000)		(0.000)		(0.000)
AR(2)		1.21		$-$0.58		0.99
		(0.227)		(0.562)		(0.324)
sargan		86.92		82.59		82.59
		(0.103)		(0.283)		(0.204)
*N*	16,050	13,815	3543	3297	16,050	13,815

***, **, * denote significance at 1%, 5%, and 10%, respectively. Z-statistics are report in parentheses. The dependent variables in columns (1), (3), and (5) are $\ln output$, $\ln EP{I_1}$, and$\ln EP{I_2}$, respectively. The dependent variable in columns (2), (4), and (6) is $\ln L$

**Table 7:** Employee heterogeneity analysis

Variables	(1)	(2)	(3)	(4)	(5)	(6)
$\ln GT$	$-$1.143***	$-$0.518***	$-$0.245	$-$0.298***	$-$0.496*	0.735**
	($-$3.30)	($-$3.11)	($-$0.14)	($-$3.98)	($-$1.67)	(2.10)
$\ln (GT)^2$	0.678***	0.160*	0.016	0.109***	0.218*	$-$0.189
	(3.36)	(1.78)	(0.02)	(3.10)	(1.72)	($-$1.02)
$\_cons$	5.171***	2.558***	3.191**	2.907***	2.335***	3.326***
	(7.00)	(5.31)	(2.30)	(7.03)	(6.42)	(6.91)
Control						
variables	Yes	Yes	Yes	Yes	Yes	Yes
Year FE	Yes	Yes	Yes	Yes	Yes	Yes
AR(1)	$-$4.92	$-$4.94	$-$5.16	$-$2.56	$-$10.69	$-$6.38
	(0.000)	(0.000)	(0.000)	(0.010)	(0.000)	(0.000)
AR(2)	$-$0.24	$-$0.80	2.56	$-$0.62	1.59	1.67
(0.809)	(0.423)	(0.111)	(0.534)	(0.111)	(0.101)	
sargan	46.66	39.76	83.67	108.32	61.08	
	(0.647)	(0.223)	(0.436)	(0.428)	(0.132)	(0.437)
*N*	2178	1995	2249	702	10,730	2365

***, **, * denote significance at 1%, 5%, and 10%, respectively. Z-statistics are reported in parentheses. Columns (1), (2), and (3) express the classification of jobs, which are production personnel, technical personnel, and other personnel, respectively. Columns (4), (5), and (6) indicate the classification of academic degrees, which are low-skilled labor, medium-skilled labor, and high-skilled labor, respectively

### Mechanism analysis

Drawing from our previous theoretical analysis, we posited that enterprise green transformation affects employment by influencing the scale of production. We use the natural logarithm of current operating revenue to represent the production scale of the enterprise ($\ln output$). Column (1) of Table 6 displays the effect of green transformation on output, revealing that the coefficient of $\ln GT$ is significantly negative, while the coefficient of $\ln (GT)^{2}$ is significantly positive. Column (2) of Table 6 shows the effect of output on employment, indicating that the coefficient of $\ln output$ is 0.066 and significant at the 1% level. These findings suggest that green transformation reduces output in the short term, which in turn inhibits employment. However, in the long term, green transformation increases employment by improving output. Therefore, enterprise green transformation has the potential to induce output growth and employment virtuous cycle.

The theoretical analysis also suggests that green transformation affects employment by influencing environmental protection investment, and this effect depends on whether it is a substitution or complementary effect. There are two ways to represent environmental protection investment: one is the expenditure related to environmental protection in the “construction in progress” account ($ln EP{I_1}$),^3^ and the other is the expenses related to environmental protection in the “administration expenses” account ($ln EP{I_2}$).^4^ Columns (3) and (5) of Table 6 present the impact of green transformation on environmental protection investment, revealing that the coefficients of *lnGT* are significantly positive. Columns (4) and (6) of Table 6 display the effect of environmental protection investment on employment, indicating that the coefficients are 0.017 and 0.028, respectively, and pass the significance test. This illustrates that environmental protection investment promotes employment, creating a factor complementary effect. In conclusion, enterprise green transformation increases employment through a factor complementary effect.

**Fig. 2:**
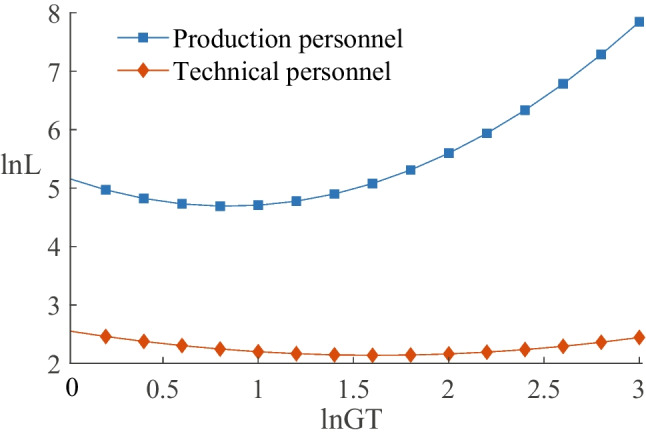
Comparison of different jobs

**Fig. 3:**
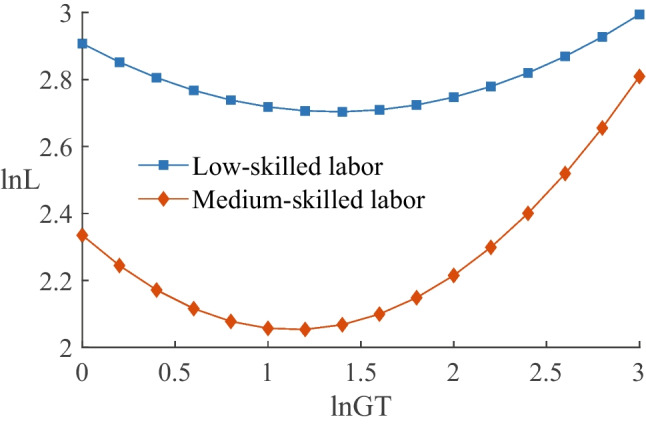
Comparison of academic degrees

### Heterogeneity analysis

#### Impact on employees with different jobs

We grouped employees into production personnel, technical personnel, and other personnel (including sales, finance, management, and administrative personnel) based on their job positions, and the regression results are reported in columns (1), (2), and (3) of Table 7. The results indicate that the coefficients of $\ln GT$ for production personnel and technical personnel are $-$1.143 and $-$0.518, respectively, while the coefficients of $\ln (GT)^{2}$ are 0.678 and 0.160, respectively. All coefficients pass the significance test, indicating a U-shaped relationship between green transformation and employment for production personnel and technical personnel. However, neither the linear nor the quadratic term of green transformation is significant for other personnel. We also compared the U-shaped curve characteristics of production personnel and technical personnel. Figure 2 shows that the employment turning point for production personnel is to the upper left of that for technical personnel, and the absolute value of the slope for production personnel is larger. These findings suggest that green transformation has a greater employment deprivation for production personnel in the short term, while the reversal of employment for technical personnel requires a higher level of green transformation to be achieved.

**Table 8:** Enterprise heterogeneity analysis

Variables	(1)	(2)	(3)	(4)	(5)	(6)	(7)
$\ln GT$	$-$0.153***	$-$0.763***	$-$0.741***	$-$0.648*	$-$0.043***	$-$0.779*	$-$0.816***
	($-$3.92)	($-$3.03)	($-$2.78)	($-$1.67)	($-$2.67)	($-$1.68)	($-$2.95)
$\ln (GT)^2$	0.083***	0.280***	290***	0.268*	0.073***	0.324*	0.332***
	(3.42)	(2.77)	0. (2.76)	(1.75)	(7.90)	(1.70)	(3.02)
$\_cons$	6.650***	9.661***	0.570**	0.778*	1.854***	0.862*	2.450***
	(18.92)	(15.92)	(2.17)	(1.69)	(3.78)	(1.65)	(6.74)
Control							
variables	Yes	Yes	Yes	Yes	Yes	Yes	Yes
Year FE	Yes	Yes	Yes	Yes	Yes	Yes	Yes
AR(1)	$-$4.68	$-$10.30	$-$4.84	$-$5.67	$-$4.46	$-$6.36	$-$6.02
	(0.000)	(0.000)	(0.000)	(0.000)	(0.000)	(0.000)	(0.000)
AR(2)	$-$1.85	1.47	1.10	0.67	0.07	1.00	0.92
	(0.164)	(0.141)	(0.269)	(0.502)	(0.944)	(0.318)	(0.356)
sargan	55.58	73.91	111.27	45.40	63.43	64.58	108.23
	(0.114)	(0.291)	(0.502)	(0.729)	(0.143)	(0.347)	(0.476)
*N*	5214	8501	4344	6967	2347	7647	6099

***, **, * denote significance at 1%, 5%, and 10%, respectively. Z-statistics are reported in parentheses. Columns (1) and (2) express the classification of ownership, which are state-owned and non-state-owned enterprises, respectively. Columns (3), (4), and (5) indicate the classification of pollution levels, which are heavy-polluting, medium-polluting and, low-polluting enterprises, respectively. Columns (6) and (7) show the classification of technology levels, which are high-tech and medium-low-tech enterprises, respectively

#### Impact on employees with different academic degrees

We classified employees into low-skilled labor, medium-skilled labor, and high-skilled labor according to their academic degrees, where employees with less than college degrees are considered low-skilled labor, employees with college and bachelor degrees are considered medium-skilled labor, and employees with master’s degrees or above are considered high-skilled labor. The results are presented in columns (4), (5), and (6) of Table 7. The coefficients of $\ln GT$ for low-skilled, medium-skilled, and high-skilled labor are $-$0.298, $-$0.496, and 0.735, respectively. The coefficients of $\ln (GT)^{2}$ are 0.109, 0.218, and $-$0.189, respectively, and are significant for all except the quadratic term for high-skilled labor. These findings suggest a U-shaped relationship between green transformation and low- and medium-skilled labor and a positive relationship with high-skilled labor. We also considered the U-shaped curve characteristics of green transformation for low- and medium-skilled labor. Figure 3 shows that the turning point for low-skilled labor is on the upper-right side of that for medium-skilled labor, while the absolute value of the slope for medium-skilled labor is larger. This illustrates that green transformation has a longer duration of employment disincentive effect on low-skilled labor and a greater negative impact on employment for medium-skilled labor in the short term.

**Fig. 4:**
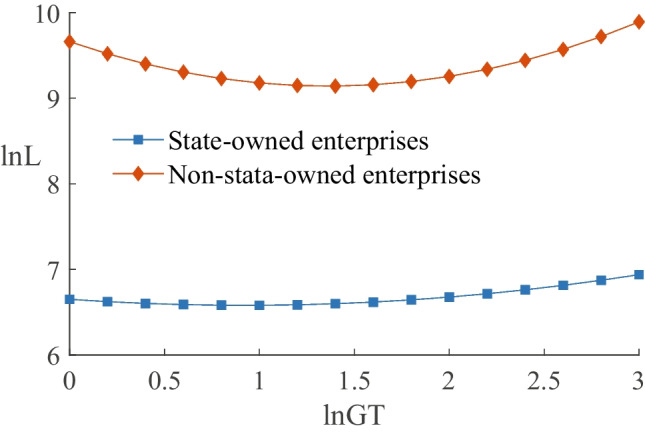
Comparison of different ownership

#### Impact on enterprises with different ownership

We conducted separate regressions for state-owned and non-state-owned enterprises based on their ownership types, and the results are reported in columns (1) and (2) of Table 8. The coefficients of $\ln GT$ are $-$0.153 and $-$0.763 for state-owned and non-state-owned enterprises, respectively, while the coefficients of $\ln (GT)^{2}$ are 0.083 and 0.280, respectively. All coefficients pass the significance test, indicating a U-shaped relationship between green transformation and employment in state-owned and non-state-owned enterprises. Next, we analyzed the U-shaped curve characteristics of employment for the green transformation process in different ownership enterprises. Figure 4 reveals that the employment turning point for state-owned enterprises is located to the below left of the turning point for non-state-owned enterprises, and the absolute value of the slope is greater for non-state-owned enterprises. Therefore, our study suggests that state-owned enterprises have a “stabilizing effect” on employment during the green transformation process and can achieve positive employment growth more quickly. Conversely, the employment of non-state-owned enterprises is more volatile, and the reform process is relatively slower.

**Fig. 5:**
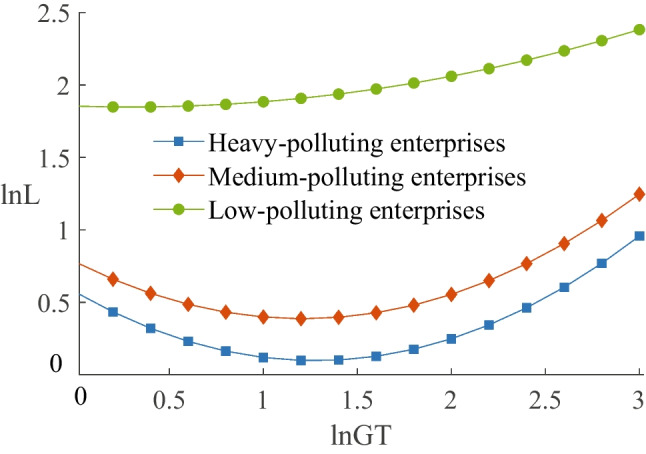
Comparison of different pollution levels

**Fig. 6:**
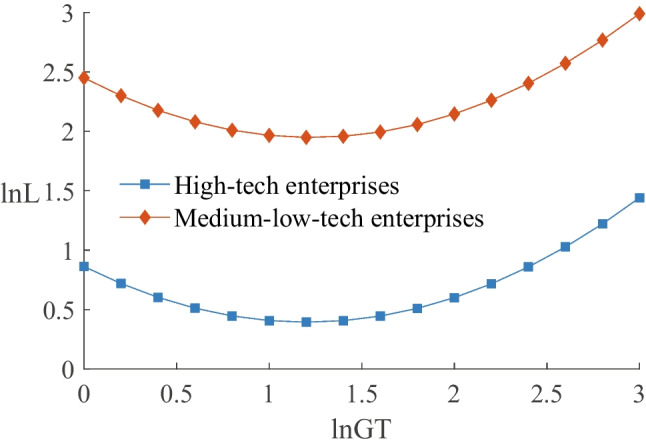
Comparison of different technology levels

#### Impact on enterprises with different pollution levels

We followed the classification of Wang and Yuan (2018) and combined the pollution classification of the “List of environmental verification industry classification management of listed companies” and “Environmental information disclosure guidelines for listed companies (2020)” to classify the samples into heavy-polluting, medium-polluting, and low-polluting enterprises based on industry pollution levels. The regression results are shown in columns (3), (4), and (5) of Table 8. The coefficients of $\ln GT$ for each pollution level are significantly negative, while the coefficients of $\ln (GT)^{2}$ are significantly positive, indicating a U-shaped relationship between green transformation and employment in enterprises with different pollution levels. Next, we compared the U-shaped curves of different pollution levels. As shown in Fig. 5, the employment turning point and the absolute value of the slope display an increasing trend as the pollution levels deepen, suggesting that more heavy-polluting enterprises are more strongly and persistently impacted by the negative effects of green transformation.

#### Impact on enterprises with different technology levels

We followed the technological classification of industries proposed by Fengju et al. (2020) and UNCTAD (2002) and classified enterprises into high-tech and medium-low-tech enterprises based on the technology levels of their respective industries. Columns (6) and (7) of Table 8 show that the coefficients of $\ln GT$ for different technology levels are significantly negative, while the coefficients of $\ln (GT)^{2}$ are significantly positive, indicating a U-shaped relationship between green transformation and employment in enterprises of different technology levels. Furthermore, Fig. 6 demonstrates that the employment turning point for high-tech enterprises is located to the below left of that for medium-low-tech enterprises, and the absolute value of the slope for medium-low-tech enterprises is larger. These findings suggest that technology levels can mitigate the negative impact of green transformation on employment and accelerate the positive employment growth effect.

## Conclusions and discussions

In the post-epidemic era, there is increasing interest in the potential of the green economy as a possible pathway out of economic distress. This has led to greater attention being paid to the link between green transformation and employment. To this end, we analyzed the impact of green transformation on labor employment and its underlying mechanism based on micro-enterprise behavior, using A-share listed companies in Shanghai and Shenzhen as our sample from 2008 to 2020. The paper draws the following conclusions.

Firstly, our analysis reveals a dynamic non-linear relationship between green transformation and employment, characterized by a U-shaped pattern. As the transformation deepens, the negative impact on employment changes to a positive one. This evidence provides support for and affirms the recent efforts toward green development. We view the initial employment deprivation effect of the green transformation as a “painful stage” that must be overcome. Only by continuously promoting green transformation and crossing the turning point of the U-shaped curve can we achieve the win-win situation of green development and employment. The turning points of the U-shaped curve for green transformation are 1.28 and 1.24, respectively. This reveals that the implementation of a single green measure is not sufficient to promote employment growth and may even have a detrimental effect on employment. In light of this, we emphasize the importance of undertaking multi-dimensional, multi-measure, and multi-directional exploration in enterprise green transformation. This conclusion holds significant meaning and highlights the need for comprehensive and diverse approaches to achieve sustainable employment outcomes.

Secondly, our analysis suggests that enterprise green transformation has the potential to induce output growth and employment virtuous cycle in the long run. Additionally, our study finds evidence of a complementary effect between environmental protection investment and labor employment in the process of green transformation. From this perspective, investments and subsidies to support green transformation may be viable policy options to address the challenges associated with the shift towards development patterns and further stimulate employment growth.

Finally, our analysis reveals that the impact of green transformation on employment varies based on different labor force and enterprise characteristics. On the one hand, the green transformation has a strong employment deprivation effect on production personnel and medium and low-skilled labor, and it takes a long time to achieve employment growth effects. However, green transformation is relatively friendly to the employment of high-skilled labor. This is confirmed by Li and Du (2022), who argue that environmental policies cause labor redistribution mainly in the form of the expansion of skilled workers and the contraction of unskilled workers, implying that skilled workers replace productive workers. Furthermore, the characteristics of labor also contribute to differences in the impact. Medium-skilled and low-skilled labor have simple and polluting jobs, which have strong substitutability,^5^ while high-skilled labor has higher adaptability and changeability (Gali , 1999; Clemens et al. , 2021). Interestingly, contrary to the conclusions of Zheng et al. ((2022), our further classification and comparison of medium-skilled and low-skilled labor suggest that, in the short term, enterprise green transformation has a greater employment deprivation effect on medium-skilled labor. This conclusion reflects the impact of China’s higher education expansion on the employment skill structure at the end of the twentieth century and also implies the reality of China’s “labor shortage” at the two ends and “employment difficulty” in the middle (Bai , 2006).

On the other hand, our study also highlights the important role of state-owned enterprises as “stabilizers” in the green transformation process. One reason for this is that state-owned enterprises are tasked with maintaining social stability and securing people’s livelihoods (Jiang and Kim , 2020). Another reason is that state-owned enterprises are more likely to receive policy incentives and financial support (García-Melón et al. , 2016), which makes them better equipped to absorb the costs of green transformation and ultimately stabilize employment and achieve positive employment growth sooner. However, we also find that heavy-polluting enterprises experience stronger employment deprivation effects during the green transformation process, as they face more government regulation and cost constraints (Hafstead and Williams III , 2018). Furthermore, our analysis demonstrates that technology can mitigate the shock of green transformation on employment and accelerate employment growth.

Based on the above conclusions, we propose the following policy suggestions:

Firstly, enterprises must implement multi-dimensional, multi-measure, and multi-directional green reform measures to continuously enhance the level of green transformation. Enterprises should simultaneously undertake comprehensive and diversified green actions, such as promoting environmental sustainability culture, enhancing green management practices, fostering green innovation, actively adopting green production methods, and improving pollutant treatment capacity. These actions aim to swiftly overcome the employment turning point and achieve a mutually beneficial situation of green development and employment.

Secondly, it is crucial for the government to develop differentiated policies that consider the distinctive characteristics and needs of various labor and enterprise groups. Green transformation can have a disproportionate impact on employment opportunities, especially for production personnel, low-skilled labor, non-state-owned, heavy-polluting, and medium-low-tech enterprises. These groups often face unique challenges and vulnerabilities. On the one hand, the government should strengthen the skill training and vocational education for low-skilled labor, so as to better meet the growing demand of enterprises for skilled labor in the context of green transformation. On the other hand, providing greater policy support and financial assistance to non-state-owned and heavy-polluting enterprises and giving them longer transition periods can be a viable strategy to mitigate the cost and risks associated with green transformation.

Thirdly, the government should enhance policy support for technological innovation within enterprises to drive transformation and ensure employment. The indirect positive effect of green transformation on employment can be achieved by enhancing the innovation capabilities of enterprises. This can help compensate for any initial employment losses during the transformation process and expedite the passage of the employment turning point.

Our study has several limitations. Firstly, we are limited by the availability of data on enterprise green behavior. Our measurement of enterprise green transformation is mainly based on the simple addition of various green measures, which may not accurately reflect the specific implementation effect of these measures and could introduce certain estimation bias. Secondly, our analysis focuses solely on the scale of employment. Given the double dilemma of “shortage of workers” and “difficulty in employment,” it is necessary to explore the structure of employment skills and the quality of employment matching in future research. Additionally, our study only includes samples of listed companies, limiting the generalizability of our conclusions to small- and medium-sized enterprises. In the future, the framework of our study can be applied to small- and medium-sized enterprises to obtain different insights.

## Data Availability

The raw data supporting the conclusions of this article will be made available by the authors, without undue reservation.

## Footnotes

1 Compared with the difference-GMM, the system-GMM can improve the estimation efficiency and estimate the coefficients of variables that do not change with time. Therefore, we choose the system-GMM method in our analysis.

2 Environmental experience of executives refers to their engagement in work related to environmental protection, such as holding positions in governmental environmental protection departments or associations, participating in environmental protection projects, obtaining environmental protection-related degrees, or patented technologies. The data used to identify environmental experience comes from the CSMAR profile files of executives.

3 To calculate the expenditure related to environmental protection in the “construction in progress” account, we use keywords such as “environmental protection,” “energy saving,” “emission reduction,” “wastewater,” “waste gas,” “solid waste,” and “low carbon” to add up the total environmental protection investment.

4 To calculate the expenses related to environmental protection in the “administration expenses” account, we use keywords such as “environmental protection,” “pollution,” “pollution treatment,” “clean production,” and “sludge” to add up the total environmental protection investment.

5 Substitutability comes from two sources. Firstly, high-skilled labor replaces low-skilled labor in the enterprise. Second, capital or technological progress replaces low-end labor demand,
